# Porcine reproductive and respiratory syndrome virus 2 (PRRSV-2) genetic diversity and occurrence of wild type and vaccine-like strains in the United States swine industry

**DOI:** 10.1371/journal.pone.0259531

**Published:** 2021-11-19

**Authors:** Mariana Kikuti, Juan Sanhueza, Carles Vilalta, Igor Adolfo Dexheimer Paploski, Kimberly VanderWaal, Cesar A. Corzo

**Affiliations:** 1 Department of Veterinary Population Medicine, University of Minnesota, Saint Paul, MN, United States of America; 2 Facultad de Recursos Naturales, Departamento de Ciencias Veterinarias y Salud Pública, Universidad Católica de Temuco, Temuco, Araucanía, Chile; 3 Upnorth Analytics, Barcelona, Spain; North Carolina State University College of Veterinary Medicine, UNITED STATES

## Abstract

Porcine reproductive and respiratory syndrome virus genotype 2 (PRRSV-2) genetic diversity in the U.S. was assessed using a database comprising 10 years’ worth of sequence data obtained from swine production systems routine monitoring and outbreak investigations. A total of 26,831 ORF5 PRRSV-2 sequences from 34 production systems were included in this analysis. Within group mean genetic distance (i.e. mean proportion of nucleotide differences within ORF5) per year according to herd type was calculated for all PRRSV-2 sequences. The percent nucleotide difference between each sequence and the ORF5 sequences from four commercially available PRRSV-2 vaccines (Ingelvac PRRS MLV, Ingelvac PRRS ATP, Fostera PRRS, and Prevacent PRRS) within the same lineage over time was used to classify sequences in wild-type or vaccine-like. The mean ORF5 genetic distance fluctuated from 0.09 to 0.13, being generally smaller in years in which there was a relative higher frequency of dominant lineage. Vaccine-like sequences comprised about one fourth of sequences obtained through routine monitoring of PRRS. We found that lineage 5 sequences were mostly Ingelvac PRRS MLV-like. Lineage 8 sequences up to 2011 were 62.9% Ingelvac PRRS ATP-like while the remaining were wild-type viruses. From 2012 onwards, 51.9% of lineage 8 sequences were Ingelvac PRRS ATP-like, 45.0% were Fostera PRRS-like, and only 3.2% were wild-type. For lineage 1 sequences, 0.1% and 1.7% of the sequences were Prevacent PRRS-like in 2009–2018 and 2019, respectively. These results suggest that repeated introductions of vaccine-like viruses through use of modified live vaccines might decrease within-lineage viral diversity as vaccine-like strains become more prevalent. Overall, this compilation of private data from routine monitoring provides valuable information on PRRSV viral diversity.

## Introduction

Viral evolution plays an important role in the ecology of infectious diseases, particularly for RNA viruses such as Porcine reproductive and respiratory syndrome virus (PRRSV), which is in the *Arteriviridae* family in the order *Nidovirales* with a genome 14.9 to 15.5kb in length [1,2]. Diversity of RNA viruses results from mutation, genomic recombination, and genome reassortment [3]. Therefore, the more opportunities the virus is given to complete transmission cycles, the higher the probability that the virus can change [4–6]. In contemporary pig production systems, PRRSV has clearly demonstrated the ability to persist in populations. The virus takes advantage of factors such as a) continuous introductions of susceptible animals to a herd and a region, b) heterogeneities in immunity due to asynchronous seroconversion of animals during an outbreak, and c) the short duration of passive immunity in young pigs, making the recently weaned or growing pigs susceptible to infection or re-infection [7,8].

During the early 1990’s, two types of PRRSV were isolated for the first time both in Europe and North America. The European strain was named the Lelystad virus and the North American strain as VR2332 [9]. Genotypical differences between the European and North American first isolates led to the classification of PRRSV into two distinct species, *Betaarterivirus suid 1* (PRRSV-1) and *Betaarterivirus suid 2* (PRRSV-2) [10]. The pairwise genetic distance between PRRSV-1 and PRRSV-2 has been described at around 35% for open reading frames 2 through 7 [11]. Since its early identification, technological development of PRRSV diagnosis has shifted towards rapid viral identification and characterization through widespread sequencing.

Nucleotide sequencing of the open reading frame 5 (ORF5) region of the viral genome has become a popular methodology to differentiate genotypes and strains, and to understand viral diversity across time. This region is of interest for being one of the most diverse regions of the PRRSV genome and for encoding the major envelope GP5 protein, which plays a role in the attachment to the target cells and to which antibodies against certain epitopes have neutralizing activity [12–15]. Based on this data, an ORF5 phylogeny-based lineage and sub-lineage classification has been proposed in which sequences were classified by comparing them to a reference sequence set with the overarching goal of describing PRRSV-2 diversity [16,17].

Additionally, PRRS is one of the main infectious diseases affecting swine, and causes an average economic loss of US$664 million per year in the US [18]. Thus, efforts in immunizing the herds have been made in the past few decades, particularly in high density swine regions. Current PRRSV vaccines main outcome is to reduce economic losses due to the disease by reducing viremia and clinical manifestations, such as respiratory signs and macro- and microscopic lung lesions compared to non-vaccinated animals rather than preventing infection [19–21]. However, the use of live vaccines implies viral replication with the potential of vaccine-derived virus shedding through the process. Still, these vaccines have the potential to reduce disease transmission [22,23].

Over the past decades, producers and veterinarians have been investing in diagnostics and sequencing as a way to further understand the epidemiology of PRRSV at both the farm and production system level. The amount of ORF5 sequencing information historically generated privately by the swine industry, if combined, has the potential of revealing the overall viral diversity of PRRSV in the major swine production areas of the United States (U.S.), which is currently poorly described. Our goal therefore was to describe PRRSV-2 genetic diversity and the frequency of vaccine-like strains in the course of over 10+ years of routine monitoring in the U.S. swine industry.

## Materials and methods

Data for this project ranged between January, 2009 to December, 2019 was obtained through the Morrison Swine Health Monitoring Project (MSHMP), which is a voluntary initiative in the U.S. that monitors PRRS occurrence in the country, while working towards foreign disease preparedness. In 2011, MSHMP began collecting both retrospective and prospective data, and currently has 37 participating production systems sharing weekly breeding herd status information of farms that account for approximately 50% of the U.S. sow population. Participants report PRRSV infection status of sow farms weekly as well as share PRRSV ORF5 sequences as a result of their outbreak investigation [24]. In addition, participants also share sequences obtained from their routine monitoring efforts in breeding, gilt developing units, growing and finishing herds. Sequences are generally obtained either directly from each MSHMP participant or from the main veterinary diagnostic laboratory where participants submit their diagnostic samples. Sequences are accompanied by farm name, date and farm type of origin (e.g. breeding or growing herd).

All sequences received for diagnosis dated from 2009 to 2019 were aligned using MAFFT (Multiple Alignment using Fast Fourier Transform) alignment on Geneious Prime 2020.2 (https://www.geneious.com). Then aligned sequences were genotyped by calculating the percent identity (e.g. proportion of nucleotides characters that match) between each sequence and the first PRRSV-2 isolated in the U.S. (VR2332) [9] and the first PRRSV-1 isolated in the Netherlands (Lelystad) [25]. Sequences that had a higher similarity to VR2332 than the Lelystad virus were then considered PRRSV-2. All PRRSV-2 sequences were then described according to their within group mean genetic distance (i.e. mean proportion of nucleotide differences within ORF5) per year, herd type, and lineage on MEGA X [26].

PRRSV-2 sequences were further classified into nine previously described phylogenetic lineages [16] and eight sub-lineages [17] for the highly diverse lineage 1 group by assigning each sequence to the lineage/sub-lineage which it was most closely related based on percent nucleotide identity to a reference set of sequences [17].

Four commercially available vaccines are utilitzed in the United States: 1) Ingelvac PRRS MLV (Boehringer Ingelheim Animal Health, Duluth, Georgia, USA)–lineage 5 (GenBank no. AF066183.4), 2) Ingelvac PRRS ATP (Boehringer Ingelheim Animal Health, Duluth, Georgia, USA)–lineage 8 (GenBank no. EF532801.1), 3) Fostera PRRS (Zoetis, Parsipanny, New Jersey, USA)–lineage 8 (GenBank no. AF494042.1), and 4) Prevacent PRRS (Greenfield, Indiana, USA)–lineage 1 (GenBank no. KU131568.1). These four vaccines were introduced into the swine market at different time point, more specifically, Ingelvac PRRS MLV, Ingelvac PRRS ATP, Fostera PRRS, and Prevacent PRRS were introduced in 1994, 2004, 2012 and 2018, respectively. Within each vaccine-associated lineage, the percent nucleotide difference between each sequence and the ORF5 sequences from the commercially available PRRSV-2 vaccines within the same lineage were plotted by year to illustrate diversification patterns of vaccine lineages and occurrence of vaccine-like isolates through time. Sequences with <5% nucleotide identity to a vaccine strain were considered vaccine-like sequences. Based on the bi-modal distribution of genetic distances from the vaccine strains, a 5% cutoff was used to classify sequences as vaccine-like or wild-type. Frequency of vaccine-like sequences by herd type were compared by chi-square. Lineage 8 wild-type sequences were also described by region and compared by Fisher exact.

## Results and discussion

A total of 27,875 PRRSV sequences from 2009 to 2019 were received from 34 participant systems. Because most sequences (26,853 out of 27,875; 96.3%) were PRRSV-2, PRRSV-1 sequences were excluded from the analysis. We also excluded 22 PRRSV-2 ORF5 sequences with incorrect initial and/or stop codons. The remaining 26,831 PRRSV-2 sequences were further analyzed.

Lineage and sub-lineages occurrence over time was similar to what was previously described with a regional subset of this dataset [17], with Lineage 1 and its sub-lineages comprising the majority of our dataset since 2010. The mean ORF5 genetic distance within each year ranged from 0.09 to 0.13 across 2009–2019 (Table 1). The mean genetic distance falls within the range expected based upon between lineage genetic distances previously described [17,27]. The yearly increase and decrease in mean genetic distance possibly explained by the change in frequency of the dominant lineages and sub-lineages over time [17] that was also observed with this dataset (S1 Fig).

**Table 1:** Within-year mean ORF5 genetic distance as proportion nucleotide differences overall and by herd type.

	2009	2010	2011	2012	2013	2014	2015	2016	2017	2018	2019
Overall												
Total sequences	1,223	1,439	1,610	2,462	2,041	2,304	3,812	3,687	3,082	3,240	1,931	
Mean distance	0.13	0.13	0.13	0.13	0.13	0.13	0.09	0.10	0.11	0.12	0.13	
Breeding												
Total sequences	285	598	603	578	429	518	1,670	937	862	791	434	
Mean distance	0.10	0.12	0.11	0.11	0.12	0.10	0.06	0.08	0.10	0.11	0.12	
Grow-finishing												
Total sequences	69	73	137	428	396	635	579	1,338	1,014	1,197	540	
Mean distance	0.11	0.10	0.11	0.12	0.12	0.12	0.10	0.09	0.10	0.10	0.11	

**Fig 1:**
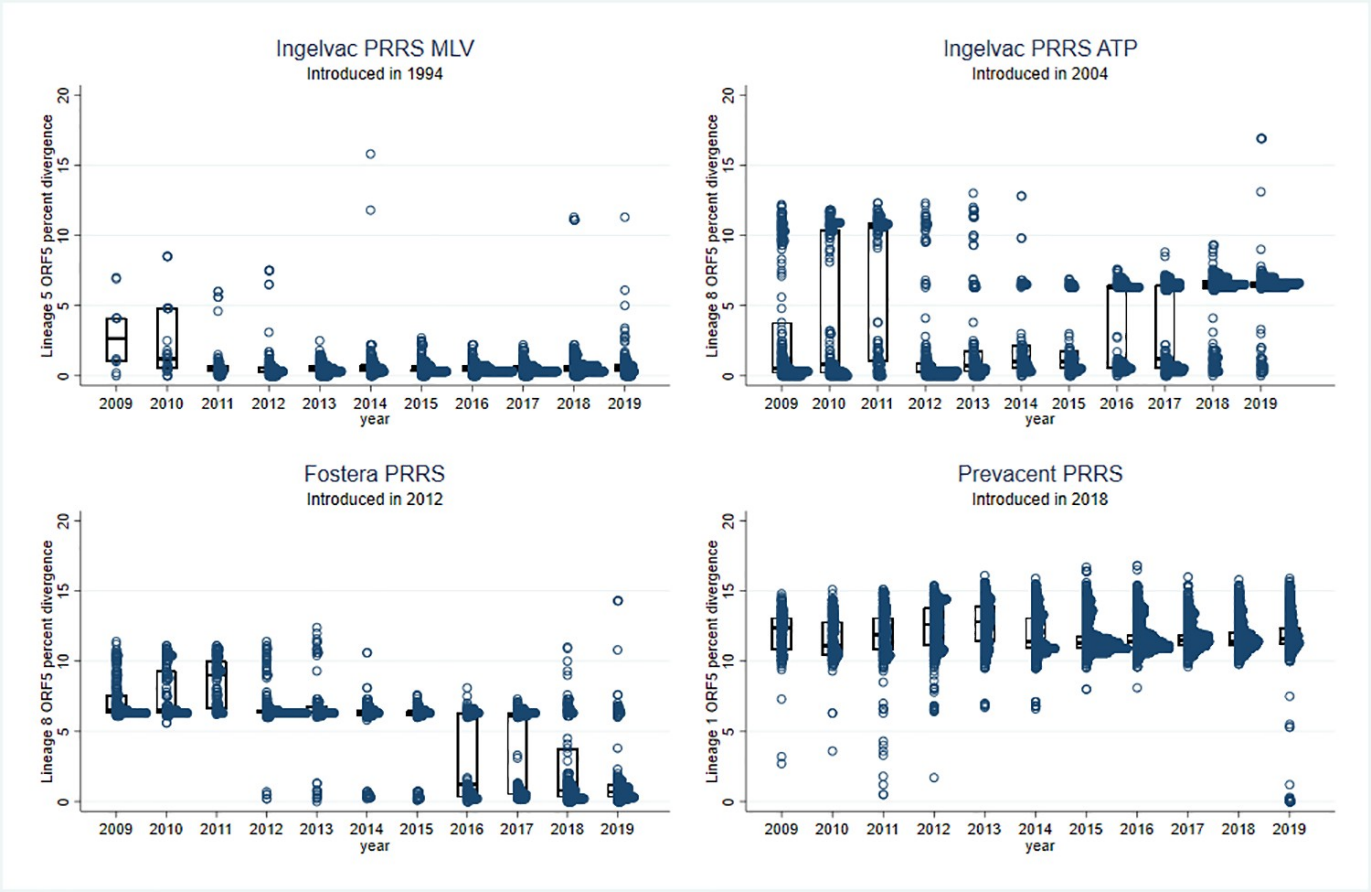
Yearly percent nucleotide differences between lineage 5 ORF5 sequences to Ingelvac PRRS MLV; lineage 8 sequences to either Ingelvac PRRS ATP or Fostera PRRS; and lineage 1 sequences to Prevacent PRRS.

Genetic distances were calculated to vaccine strains for sequences within the same lineage as a modified live vaccine. A total of 18,502 lineage 1 sequences (including all sub-lineages) were compared to Prevacent PRRS, 2,408 lineage 5 sequences were compared to Ingelvac PRRS MLV, and 2,313 lineage 8 sequences were compared to Ingelvac PRRS ATP and Fostera PRRS. The overall frequency of other lineages within this database is illustrated in S1 Fig. The percent nucleotide difference between each sequence and the vaccine per year is described in Fig 1. Most lineage 5 sequences (2,383/2,408; 99.0%) present on our dataset were less than 5% different from Ingelvac PRRS MLV in the ORF5 region throughout the entire study period.

For lineage 8, however, we found a bimodal distribution of nucleotide differences when compared to Ingelvac PRRS ATP, with one subgroup <5% different from the vaccine strain (1,267/2,313; 54.8%) and another subgroup 5.6–16.9% different throughout the entire study period. This finding is similar to what was observed using a global PRRSV-2 dataset that classified sequences into vaccine-like or wild-type based on their relative distances to the parental or vaccine strains, in which they found 80% and 60% of sub-lineages 5.1 and 8.9, respectively, to be vaccine-related [28].

When comparing lineage 8 sequences to Fostera PRRS, we observed an increasing proportion of sequences <5% different to the vaccine since 2012, which is the year when the vaccine was introduced onto the market. After that, a bimodal distribution of nucleotide differences was found, with one subgroup <5% different from the vaccine strain and another subgroup 5.8–14.3% different. Of note, ORF5 sequences for Ingelvac PRRS ATP and Fostera PRRS have a percent nucleotide identity to each other of 93.7%. Using <5% nucleotide difference as a cut-off for vaccine-like sequence classification, 62.9% (383/609) of all lineage 8 sequences up to 2011 were Ingelvac PRRS ATP-like while the remaining would be considered wild-type. However, from 2012 onwards, 51.9% (884/1,704) were Ingelvac PRRS ATP-like, 45.0% (766/1,704) were Fostera PRRS-like, and only the remaining 3.2% (54/1,704) would be considered wild-type. Information on either state or region was available for 40.68% (941/2,313) of the L8 sequences, most of which were from the Midwest (459, 48.78%) and the South (440, 46.76%). Among sequences from the Midwest, 338 (73.64%) were Ingelvac PRRS ATP-like, 99 (21.57%) were Fostera PRRS-like, and 22 (4.79%) were wild-type sequences. In the south, 356 (80.91%) were Fostera PRRS-like, 82 (18.64%) were Ingelvac PRRS ATP-like, and 2 (0.45%) were wild-type. Wild-type occurrence was significantly lower in the South than in the Midwest (Fisher exact *p*<0.0001). Factors involved in the reduction in Lineage 8 wild-type occurrence are currently unknown. One hypothesis is that the introduction of Fostera PRRS or the combination of more than one available commercial vaccine of the same lineage contributed to the reduction. However, it is important to also consider the broader context of PRRSV in the United States, where Lineage 1A emerged in 2014 and quickly became the most prevalent PRRS lineage [17]. A more likely explanation is that this emergence led to viral competition playing an important role in the decrease of Lineage 8 wild-type viruses.

The constant reintroduction of a live modified virus through vaccines may have decreased the detected viral diversity for lineages 5 and 8 (Table 2), favoring vaccine-like strains. However, we were not able to observe this effect for lineage 1, which was the most prevalent lineage in the dataset. A possible explanation is that the lineage 1 vaccine (Prevacent PRRS) was only introduced in 2018. The percentage of lineage 1 sequences that were <5% different from the strain used in the Prevacent PRRS vaccine was 0.1% (12/17,224) prior to its introduction to the U.S. market in 2018, whereas 1.7% (21/1,266) were Prevacent PRRS-like in 2019. This indicates an increase in the frequency of Prevacent PRRS-like sequences, following the same pattern as other modified-live vaccines. Using the <5% difference to any vaccine sequence as a vaccine-like strain definition, we found a total of 23.8% (6,372/26,831) of our entire dataset is comprised of vaccine-like sequences, with an average of 23.7% per year ranging from 13.5% to 34.5% (Table 3).

**Table 2:** Within-year mean ORF5 genetic distance as proportion nucleotide differences by lineage.

	2009	2010	2011	2012	2013	2014	2015	2016	2017	2018	2019
Lineage 1											
Total sequences	380	648	964	1762	1370	1699	3169	2914	2174	2156	1266
Mean distance	0.10	0.09	0.10	0.11	0.12	0.11	0.07	0.08	0.09	0.10	0.11
Lineage 5											
Total sequences	10	31	92	172	259	205	289	349	321	458	222
Mean distance	0.05	0.04	0.01	0.01	0.01	0.01	0.01	0.01	0.01	0.01	0.01
Lineage 8											
Total sequences	257	203	149	245	151	136	138	198	245	281	310
Mean distance	0.05	0.07	0.09	0.03	0.04	0.03	0.03	0.04	0.04	0.03	0.03

**Table 3:** Frequency of PRRSV-2 sequences similar to vaccine strains at a >5% nucleotide identity cutoff overall and by herd type between 2009 and 2019 in the U.S.

	2009	2010	2011	2012	2013	2014	2015	2016	2017	2018	2019
Overall											
Any vaccine-like	26.49% (324/1,223)	16.33% (235/1,439)	13.48% (217/1,610)	21.28% (524/2,462)	27.63% (564/2,041)	23.96% (552/2,304)	16.34% (623/3,812)	20.61% (760/3,687)	27.55% (849/3,082)	32.62% (1,057/3,240)	34.54% (667/1,931)
Lineage 5 Ingelvac PRRS MLV-like	80.00% (8/10)	90.32% (28/31)	95.65% (88/92)	96.51% (166/172)	100% (259/259)	99.02% (203/205)	100% (289/289)	100% (349/349)	100% (321/321)	98.91% (453/458)	98.65% (219/222)
Lineage 8 Ingelvac PRRS ATP-like	75.49% (194/257)	63.05% (128/203)	40.94% (61/149)	86.94% (213/245)	91.46% (123/151)	79.41% (108/136)	79.71% (110/138)	47.98% (95/198)	55.51% (136/245)	23.13% (65/281)	10.97% (34/310)
Lineage 8 Fostera PRRS-like	0.00% (0/257)	0.00% (0/203)	0.00% (0/149)	2.04% (5/245)	7.95% (12/151)	17.65% (24/136)	20.29% (28/138)	52.02% (103/198)	44.49% (109/245)	75.80% (213/281)	87.74% (272/310)
Lineage 1 Prevacent PRRS-like	0.53% (2/380)	0.15% (1/648)	0.83% (8/964)	0.06% (1/1,762)	0.00% (0/1,370)	0.00% (0/1,699)	0.00% (0/3,169)	0.00% (0/2,914)	0.00% (0/2,174)	0.00% (0/2,156)	1.66% (21/1,266)
Breeding											
Any vaccine-like	4.21% (12/285)	2.68% (16/598)	1.33% (8/603)	10.03% (58/578)	19.81% (85/429)	6.18% (32/518)	6.29% (105/1,670)	15.8% (148/937)	13.46% (116/862)	20.35% (161/791)	27.88% (121/434)
Lineage 5 Ingelvac PRRS MLV-like	100% (2/2)	100% (6/6)	0.00% (0/2)	100% (25/25)	100% (63/63)	100% (17/17)	100% (75/75)	100% (80/80)	100% (31/31)	92.31% (60/65)	100% (42/42)
Lineage 8 Ingelvac PRRS ATP-like	100% (8/8)	100% (8/8)	100% (8/8)	95.65% (22/23)	0.00% (0/2)	12.5% (1/8)	100% (4/4)	0.00% (0/24)	0.00% (0/42)	9.21% (7/76)	0.00% (0/66)
Lineage 8 Fostera PRRS-like	0.00% (0/8)	0.00% (0/8)	0.00% (0/8)	0.00% (0/23)	100% (2/2)	75.00% (6/8)	0.00% (0/4)	100% (24/24)	100% (42/42)	90.79% (69/76)	100% (66/66)
Lineage 1 Prevacent PRRS-like	0.00% (0/28)	0.00% (0/242)	0.00% (0/360)	0.00% (0/463)	0.00% (0/332)	0.00% (0/480)	0.00% (0/1,552)	0.00% (0/788)	0.00% (0/740)	0.00% (0/622)	1.26% (4/317)
Grow-finishing											
Any vaccine-like	5.8% (4/69)	12.33% (9/73)	22.63% (31/137)	23.13% (99/428)	30.3% (120/396)	24.88% (158/635)	27.29% (158/579)	20.33% (272/1,338)	33.63% (341/1,014)	39.43% (472/1,197)	41.67% (225/540)
Lineage 5 Ingelvac PRRS MLV-like	NA	NA	90.00% (18/20)	91.38% (53/58)	100% (59/59)	100% (72/72)	100% (88/88)	100% (142/142)	100% (198/198)	100% (257/257)	97.10% (67/69)
Lineage 8 Ingelvac PRRS ATP-like	100% (4/4)	NA	100% (5/5)	100% (10/10)	50.00% (5/10)	0.00% (0/7)	32.00% (8/25)	0.00% (0/60)	6.00% (3/50)	0.00% (0/67)	0.81% (1/124)
Lineage 8 Fostera PRRS-like	0.00% (0/4)	NA	0.00% (0/5)	0.00% (0/10)	40.00% (4/10)	100% (7/7)	68.00% (17/25)	100% (60/60)	94.00% (47/50)	100% (67/67)	99.19% (123/124)
Lineage 1 Prevacent PRRS-like	0.00% (0/6)	0.00% (0/49)	0.00% (0/94)	0.00% (0/302)	0.00% (0/263)	0.00% (0/455)	0.00% (0/418)	0.00% (0/1,060)	0.00% (0/643)	0.00% (0/724)	3.99% (13/326)

A total of 52.6% (n = 14,111) of the submissions for sequencing contained information on herd type, with 54.6% and 45.4% of the sequences originating from breeding (n = 7,705) and grow-finish herds (n = 6,406), respectively. A spike in breeding farm sampling was observed in 2015 and likely represents more intense monitoring and within farm outbreak detections due to a nation-wide PRRSV outbreak attributed to a 1-7-4 RFLP-type PRRSV reported in that year [29]. We found that the mean ORF5 genetic distance for each year amongst breeding herds ranged from 0.06 in 2015 to 0.13 in 2010, 2013, and 2019. Amongst grow-finishing herds, it ranged from 0.09 in 2016 to 0.12 in 2012–2014 (Table 1). Similarly, the fluctuation of the yearly mean genetic distance also corresponds to the frequency of dominant lineages in both the breeding and grow-finishing herds, tending to be smaller when there is a higher relative frequency the dominant lineage and smaller when the frequency of the dominant lineage is lower (S1 Fig). A total of 11.2% (862/7,705) sequences from breeding herds would have been considered vaccine-like with a <5% cutoff, averaging 11.6% per year (range 1.3%- 27.9%). On the other hand, 29.5% (1,889/6,406) of sequences from grow-finishing sites would have been classified as vaccine-like with the same criteria (average per year: 25.6%; range: 5.8%-41.7%) (Table 3). Thus, vaccine-like sequences were overall more frequent amongst grow-finishing than breeding herds (chi-square *p*<0.0001). However, only a fraction of sequences had herd type information and sequences from grow-finishing sites were much less frequent before 2012, which could have introduced some bias and limit interpretation of these results.

PrimePac PRRS (lineage 7 –GenBank no. AF066384.1), a vaccine introduced in the U.S. market in 1996, was not included in this analysis since a total of two lineage 7 sequences were present in this dataset. We also did not take into account market shares of each vaccine, which correlates to vaccine usage in specific farm types in the field. Additionally, vaccine strains were compared only to sequences within the same lineage to illustrate its potential impact on lineage diversity. However, the decision of which vaccine will be used is not necessarily lineage-based, as PRRSV lineage classification has only recently gained traction for routine diagnosis and vaccines show variable heterologous protection, particularly in reducing clinical signs and lesions against unrelated isolates [30–32]. We decided to use an arbitrary <5% nucleotide identity to a vaccine-strain as cutoff to define vaccine-like sequences instead of their relative distances to the parental or vaccine strains, the latter of which could be interpreted as a metric of the overall divergence of the virus. However, the use of distance to a parental (or anchor) strain is heavily dependent on the anchors used, which might change between different research groups or even over time, as more reference strains are added throughout the years. The arbitrary <5% cutoff allows more reproducibility, although it might not necessarily represent a clinically or immunologically relevant criteria, nor does it necessarily represent the evolutionary relationships between strains. Additionally, vaccine-derived and wild parental viruses are genetically indistinguishable, thus the use of the term vaccine-like instead of vaccine-derived terminology throughout this manuscript. Another layer of complexity added to the interpretation of field genetic diversity is the unknown criteria involved in strain selection for vaccine production that are not usually disclosed by manufacturers. However, personal communications suggests that non-circulating strains are preferable to distinguish between wild-type and vaccine shedding more easily.

Although whole genome sequencing is expanding, currently, it is not performed as a routine surveillance tool in the swine industry. ORF5 sequencing remains the industry standard, thus, still more accurately represents the viral population circulating in the United States. Still, by not looking at the full genome, recombination outside ORF5 may have been missed and it might limit the interpretation of the overall viral genetic diversity.

There are several possible implications of a high frequency of vaccine-like viruses sequenced from field samples. First, it is unknown whether this represents vaccine shedding or if primer homology and viral concentration in a sample when co-infection between a vaccine and wild-type strain is present, for example, might favor sequencing of one strain over another, which would complicate diagnosis and hinder a more comprehensive understanding of PRRSV diversity in the field. Still, concerns about vaccine strain evolution in the field, potential recombination with wild-type viruses and vaccine-like strains causing clinical disease might arise. On the other hand, given that vaccines have been associated with decreased clinical manifestations [33,34], the decline in wild-type strains in favor of vaccine-like viruses within the same lineage might be beneficial in terms of predictability of PRRS economic impact. Further studies to investigate these issues are warranted.

## Conclusions

We observed a mean ORF5 genetic distance fluctuating within the expected range throughout the study period according to the frequency of the predominant lineage, regardless of herd type. Vaccine-like sequences comprised about one fourth of the sequences obtained through routine monitoring for PRRS during 2009–2019, with a slight increase in frequency in 2018 and 2019. Lastly, we found a decreased viral diversity within lineage in favor of the detection of vaccine-like strains after the introduction of commercial live modified virus vaccines in the U.S. market. Whether this represents that vaccine usage contributes to the reduction of wild-type strains in the field or that a vaccine strain is favored in the sequencing of vaccine and wild-type co-infected samples remains to be elucidated.

## Supporting information

S1 Checklist STROBE statement—checklist of items that should be included in reports of observational studies. (DOC)

S1 Fig Relative frequency of PRRSV lineages/sub-lineages over time overall (A), in breeding herds (B) and in grow-finishing herds (C).
(TIF)
