# Supplementary figures and images for: Porcine reproductive and respiratory syndrome virus 2 (PRRSV-2) genetic diversity and occurrence of wild type and vaccine-like strains in the United States swine industry
Source: PLoS One. 2021 Nov 19;16(11):e0259531. doi: 10.1371/journal.pone.0259531 (PMC8604284; doi:10.1371/journal.pone.0259531)

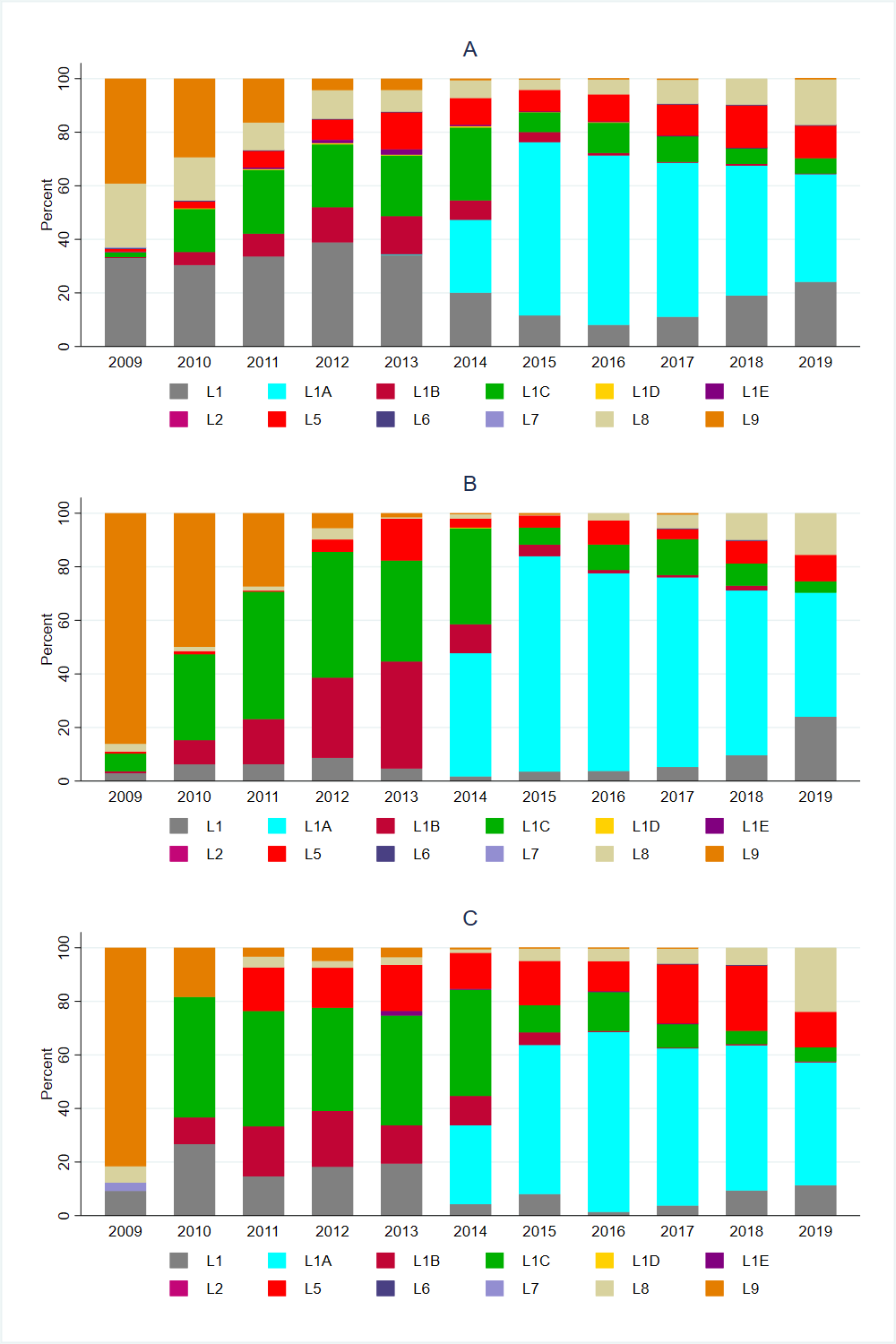

Supplement: S1 Fig — Relative frequency of PRRSV lineages/sub-lineages over time overall (A), in breeding herds (B) and in grow-finishing herds (C). (TIF) [file pone.0259531.s002.tif]
